# Clinical analysis of a case of neonatal exfoliative esophagitis in an 18-day-old neonate

**DOI:** 10.1186/s12887-019-1839-2

**Published:** 2019-11-27

**Authors:** Zu-Qin Yang, Jing-Yun Mai, Min-Li Zhu, Xiu-Man Xiao, Xiao-Xiao He, Shang-Qin Chen, Zhen-Lang Lin

**Affiliations:** Department of Neonatology, The Second Affiliated Hospital and Yuying Children’s Hospital of Wenzhou Medical University, No. 109 of Xue yuan west Street, Lucheng District, Wenzhou, 325027 China

**Keywords:** Exfoliative esophagitis, Mucosa, Pathology, Endoscopy, Neonates

## Abstract

**Background:**

This study aims to provide guidance for clinical work through analysis of the clinical characteristics, endoscopic and pathological manifestations, diagnosis, and treatment of an 18-day-old neonate with exfoliative esophagitis.

**Case presentation:**

The patient presented with vomiting but the parents did not pay too much attention. The pathological report revealed numerous fibrinous exudative necrotic, and inflammatory cells, as well as a small amount of squamous epithelium. Furthermore, milk allergy factors were considered. Conservative treatments, such as fasting, acid suppression, mucosal protection, parenteral nutrition, and the replacement of anti-allergic milk powder were given. Thereafter, endoscopic examination revealed that the patient returned to normal, and was discharged after 21 days.

**Conclusions:**

Exfoliative esophagitis has multiple causes; and has characteristic clinical and endoscopic manifestations. Endoscopic examination after 18 days presentation and conservative therapy revealed that the esophagus had returned to a normal appearance and the patient was discharged. Following discharge, the parents were advised to feed the patient ALFERE powder. Attention should be given to the timely detection of complications and corresponding treatment.

## Introduction

Exfoliative esophagitis is also known as superficial exfoliative esophagitis of the esophageal lumen and esophageal mucosal lumen exfoliation [1]. The clinical incidence of epidermic exfoliative esophagitis (EEE) in children is very low. Only one Chinese patient with EEE has been reported. This patient was a 13-year-old child with herpetic dermatitis, accompanied by esophageal mucosal exfoliation [2]. To the best of our knowledge, this is the first case report of exfoliative esophagitis in a neonate.

## Case presentation

The patient was male who was born in January 19th from Guizhou Province, China; and was born from a G2P2 mother. His gestational age was 36^+ 6^ weeks, his birth weight was 3.15 kg, and he was breastfed after birth. Prior to being admitted and treated in our department on 6th February, 2018; the patient had received hospital treatment for 1 day, for cough and vomiting, with no significant improvement.

### Physical examination results

**Fig. 1:**
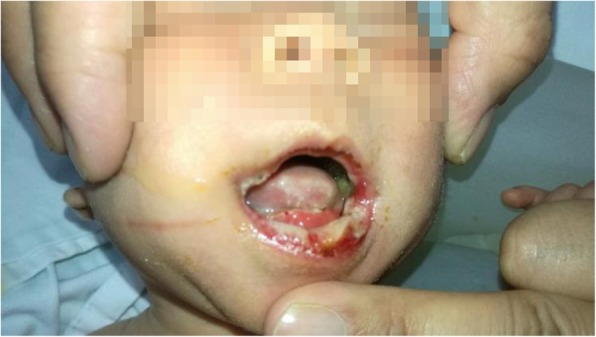
Severe ulceration of the lips

Temperature (T), 36.7 °C; pulse rate (P), 140/min; respiratory rate (R), 38/min; blood pressure (BP), 98/44 mmHg; weight (Wt), 3.41 kg. No rash, petechiae or hematomas were presented on the skin; no jaundice was observed, but the body of the tongue was covered with a yellow membrane (Fig. 1), and ulcers could be observed on the edge. The patient’s breathing was steady, the respiratory sound of the lungs was good and symmetrical but mixed with a slight phlegm sound from the throat. The heart sounds were regular and normal without any murmur. The abdomen was flat and soft, no mass was found. The liver and spleen were not enlarged. The muscle tension was in accordance with the gestational age.

### Laboratory examination results

Routine blood test: white blood cell count, 5.2 × 10^9^/l; neutrophil ratio, 0.410; haemoglobin, 126 g/l; platelet count, 288 × 10^9^/l. Ultrasonography of the pylorus was normal. Esophagography revealed no obvious abnormal x-ray-displayed lesions. Hospital day 4: C-reactive protein (CRP), 55 mg/l (normal limits is < 8 mg/l); Hospital day 5: CRP, 47 mg/l; Hospital day 6: CRP, 28 mg/l. Total IgE was 0.19 IU/ml (normal limits is 0-100 IU/ml).

**Fig. 2:**
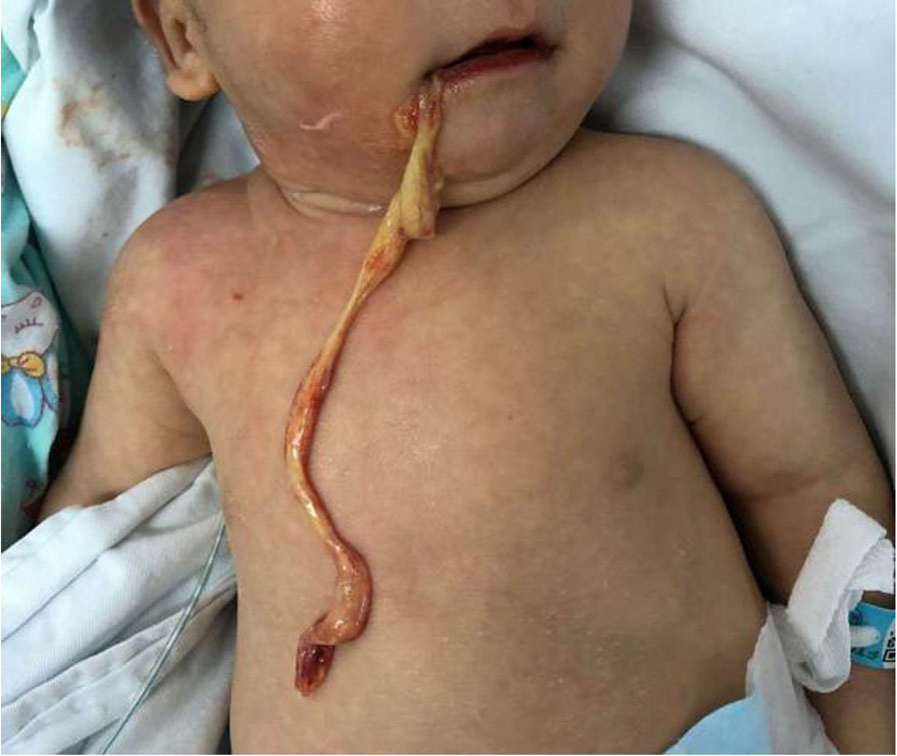
Regurgitation of the esophageal mucosal exfoliation

Following admission to our department, the patient had occasional cough and poor sucking ability. Nasogastric feeding was provided in place of oral administration of formula milk. However, the patient continued to exhibit symptoms of vomiting. Vomiting occurred on four occasions; the volumes varied and consisted of white undigested milk sample and a small amount of yellow-green mucus. Hospital day 2, 22:00: low grade fever (38 °C). Hospital day 3, 03:00: the patient vomited a strip of yellow-coloured tissue (Fig. 2). The strip, which was approximately 20 cm long, was sent for pathological examination. In addition, the patient had obvious oral ulcers, which bled when touched. Some yellowish-white membranes were attached to both buccal mucosas. CRP afterward: 27 mg/l. The patient underwent fasting, received fluid replacement, proton pump inhibitors for acid suppression, and an anti-infection injection of sulperazone (cefoperazone sodium and sulbactam sodium for injection, Pfizer pharmaceuticals LTD, Beijing). The pathology on hospital day 6 revealed numerous fibrinous exudative necrosis and inflammatory cells, a small amount of squamous epithelium, and the CRP increased to 55 mg/l. Because of the increase in CRP and the low grade fever, we decided to place the patient on a broader spectrum antibiotic regimen including tienan and vancomycin.

Paediatric surgery consultation combined with pathological reports led to the diagnosis of EEE. After 7 days of fasting, CRP level (3 mg/l) improved, and the patient was fed with milk, supplemented with vitamin B2 and zinc gluconate. The blood allergy test revealed that the patient had grade 2 allergy to milk powder; subsequently, the patient was fed with ALFERE milk powder (Nestle, Vevey, Switzerland) instead. The esophagogastroscopy revealed that the normal structures of the esophagus and stomach recovered. Hospital day 22: the patient was discharged.

## Discussion and conclusions

At present EEE has no clear etiology and most cases are in adults. Some of these cases are caused by drugs, such as exfoliative esophagitis induced by sunitinib [3], dabigatran [4] and imatinib [5]; while other cases are caused by autoimmune diseases, such as vulgaris pemphigus exfoliative esophagitis [6] and epidermolysis bullosa combined with autoantibodies against p200 pemphigus antigen. Therefore, these are correlated with exfoliative esophagitis [7]. Some cases of EEE are early exfoliative esophagitis after autologous peripheral blood stem cell transplantation [8], while some cases are caused by ingesting food that was too hot [9]. There are also patients with hepatitis B cirrhosis complicated by exfoliative esophagitis [10].

The main cause of EEE may be correlated to damage to the mucosa of the esophageal wall caused by food. The pathogenesis of EEE is due to loosen connection between the esophageal submucosa and the muscular layer. The injury causes hematoma to form easily, and this gradually increases and results in submucosal dissection. The mucous membrane on the surface of the hematoma decays and ruptures due to factors such as poor blood supply, erosion from reflux gastric juice, friction from coarse food, and when vomiting membrane or hematemesis have occurred.

Pathological examination revealed that EEE manifested as a layer of exfoliate squamous epithelium without any evidence of exfoliation of the underlying esophageal muscle layers or any evidence of esophageal perforation. The main clinical manifestations are pain, nausea and vomiting after eating, and often the patient vomits bright red blood, although our patient had no obvious blood in the vomitus. Typically, patients vomit white tubular membranes, which vary in length. Furthermore, patients may have associated chest pain and dysphagia. Some atypical patients do not vomit tubular membranes and, thus, were often mis-diagnosed.

Gastroscopy has revealed evidence of esophageal hematoma in some patients that was limited to the mucosa with preservation of the entire muscular wall of the esophagus and no evidence of esophageal perforation. Therefore, re-epithelialization of the lumen occurs with no esophageal stenosis. Rigid tube esophagoscopy increases the patients’ suffering and can cause further injury to the esophagus, it is therefore, not performed unless it is highly likely that esophageal foreign bodies are present. Flexible tube gastroscopy is an effective approach to diagnose this disease.

The patient had a grade-two allergy to milk powder, breast milk, or ordinary milk powder. These can cause edema induced by the allergic reaction of the esophageal mucosa, which may aggravate injury and exfoliation of the mucosa during vomiting. The main treatment method is fasting. The patient was given proton pump inhibitors for 2–4 weeks and could additionally receive antibiotics. The prognosis of the disease is good.

## Data Availability

All data generated or analysed during this study are included in this published article [and its supplementary information files].

## Abbreviations

CRP: C-reactive protein
EEE: Epidermic exfoliative esophagitis
Hb: Hemoglobin
Neu: Neutrophil ratio
PLT: Platelet count
WBC: White blood cell count
